# 953 Global Disparities in Burns: Analyzing Outcomes from the WHO Global Burn Registry Across Resource Settings

**DOI:** 10.1093/jbcr/iraf019.484

**Published:** 2025-04-01

**Authors:** Daniel Najafali, Saeid Rezaei, Hilary Liu, José Arellano, Erik Reiche, Quincy Tran, Sameer Patel, Peter Nthumba, Victor Stams, Francesco Egro

**Affiliations:** Carle Illinois College of Medicine; University of Pittsburgh Medical Center; University of Pittsburgh Medical Center; University of Pittsburgh Medical Center; University of Pittsburgh Medical Center; University of Maryland School of Medicine; Fox Chase Cancer Center; Vanderbilt University Medical Center; Carle Foundation Hospital; University of Pittsburgh Medical Center

## Abstract

**Introduction:**

Burn injuries remain a significant global health burden, particularly in lower-resource settings. Understanding disparities in outcomes between low-resource and high-resource regions is needed to inform interventions. We hypothesize that burns managed in low-resource countries are an independent risk factor for mortality.

**Methods:**

We queried the World Health Organization (WHO) Global Burn Registry (GBR) from inception to September 2024. Individuals were stratified based on care in low-resource (LRC) and high-resource countries (HRC). Descriptive statistics between cohorts (LRC vs. HRC) were leveraged to summarize demographics, burn characteristics, facility resources, and hospital care outcomes. Multivariable logistic regression analysis was performed for mortality while controlling for confounding factors.

**Results:**

There were 9,274 cases with 4,169 (45%) managed in LRC and 5,105 (55%) in HRC. The median age at time of burn injury was 24 years (IQR: 4-40 years). The male population was 54% in LRC and 65% in HRC (P< 0.001). The median total body surface area (TBSA) burned was higher in LRC (20%, IQR: 10-40%) compared to HRC (15%, IQR: 5-25%, P< 0.001). Inhalation injury was also more prevalent in LRC (23%) than in HRC (9%, P< 0.001). Both the median Baux and modified Baux scores were higher for LRC, 50 and 52, respectively, compared to HRC, 41 and 42, respectively (P< 0.001). Flame injuries were significantly higher in LRC (56% vs. 43% HRC, P< 0.001). There was a significantly higher proportion of head and neck, trunk, and lower extremity burn injuries in LRC (P< 0.001). Critical care resources, computer and internet access, operating room availability, rehabilitation capabilities, and specialist capabilities were significantly lower in LRC (P< 0.001). A higher proportion of patients in HRC underwent surgery (61% vs. 40% LRC, P< 0.001). Discharge was significantly higher (82% vs. 59%) in HRC and mortality was significantly lower (11%) compared to LRC (29%, P< 0.001). After adjusting for demographics and burn-related factors, management in an LRC significantly increased the odds of mortality (OR 2.69, 95%CI 2.25-3.24, P< 0.001).

**Conclusions:**

Individuals treated in LRC had higher mortality, even after adjusting for demographic and burn injury-related characteristics. These findings underscore the need for greater access to specialized care and targeted interventions in low-resource settings to improve burn care.

**Applicability of Research to Practice:**

Significant disparities in burn care exist between low-resource and high-resource settings, emphasizing the urgent need for targeted interventions. The findings suggest that increasing access to specialized burn care, critical care resources, and surgical interventions in LRC can be potential avenues to reduce mortality rates.

**Funding for the Study:**

N/A

